# Designing Fast-Dissolving Orodispersible Films of Amphotericin B for Oropharyngeal Candidiasis

**DOI:** 10.3390/pharmaceutics11080369

**Published:** 2019-08-01

**Authors:** Dolores R. Serrano, Raquel Fernandez-Garcia, Marta Mele, Anne Marie Healy, Aikaterini Lalatsa

**Affiliations:** 1 Department of Pharmaceutics and Food Technology, School of Pharmacy, Universidad Complutense de Madrid, Plaza Ramon y Cajal s/n, 28040 Madrid, Spain; 2 Instituto Universitario de Farmacia Industrial (IUFI), School of Pharmacy, Universidad Complutense de Madrid, Avenida Complutense, 28040 Madrid, Spain; 3 School of Pharmacy and Biomedical Sciences, University of Portsmouth, St. Michael’s Building, White Swan Road, Portsmouth PO1 2DT, UK; 4 Synthesis and Solid State Pharmaceutical Centre, School of Pharmacy and Pharmaceutical Sciences, Trinity College Dublin, Dublin 2, Ireland

**Keywords:** orodispersible films, fast-dissolving films, micelles, amphotericin B, fungal infections

## Abstract

Amphotericin B possesses high activity against *Candida* spp. with low risk of resistance. However, Amphotericin B’s high molecular weight compared to other antifungal drugs, such as miconazole and clotrimazole, and poor water solubility hampers its efficacy at the physiological conditions of the oropharyngeal cavity (saliva pH, limited volume for dissolution) and thereby limits its clinical use in oropharyngeal candidiasis. We have prepared fast-dissolving orodispersible films with high loading (1% *w/w*) using solvent casting that enables amphotericin B to remain solubilised in saliva in equilibrium between the monomeric and dimeric states, and able to produce a local antifungal effect. Optimisation of the amphotericin B-loaded orodispersible films was achieved by quality by design studies combining dextran and/or maltodextrin as dextrose-derived-polymer film formers with cellulose-derived film formers (hydroxypropylmethyl/hydroxypropyl cellulose in a 1:4 weight ratio), sorbitol for taste masking, microcrystalline cellulose (Avicel 200) or microcrystalline cellulose-carboxymethylcellulose sodium (Avicel CL-611) for enhancing the mechanical strength of the film, and polyethylene glycol 400 and glycerol (1:1 *w/w*) as plasticizers. The optimised amphotericin B orodispersible films (containing 1% AmB, 25% dextran, 25% maltodextrin, 5% sorbitol, 10% Avicel 200, 10% polyethylene glycol 400, 10% glycerol, 3% hydroxypropylmethyl cellulose acetate succinate, 12% hydroxypropyl cellulose) possessed a fast disintegration time (60 ± 3 s), quick release in artificial saliva (>80% in 10 min), high burst strength (2190 mN mm) and high efficacy against several *Candida* spp. (*C. albicans*, *C. parapsilosis* and *C. krusei*) (>15 mm inhibition halo). Amphotericin B orodispersible films are stable for two weeks at room temperature (25 °C) and up to 1 year in the fridge. Although further toxicological and in vivo efficacy studies are required, this novel Amphotericin B orodispersible films is a promising, physicochemically stable formulation with potential wide application in clinical practice, especially for immunocompromised patients suffering from oropharyngeal candidiasis.

## 1. Introduction

Fungal infections of the oral cavity are opportunistic, usually caused by *Candida albicans*, and occur more frequently in patients that are immunocompromised (e.g., HIV, cancer patients), diabetics or having predisposing factors such as antibiotic and corticosteroid therapy, poor buccal hygiene and ill-fitted dentures. Local therapy of oral and pharyngeal candidiasis is desirable as it avoids adverse effects linked to systemic antifungal use [1]. A major challenge to effective local treatments remains the low volume for dissolution and need for rapid permeability of the oropharyngeal cavity in order to deliver adequate drug concentrations for local action. The majority of formulations rely on oral suspensions due to the poor aqueous solubility of most antifungals that are swished around in the mouth for a few seconds, gargled, and swallowed or spat out. This short contact time with the oral mucosa requires a readily available and solubilised drug to exert an antifungal effect.

Amphotericin B (AmB) is a broad spectrum antifungal effective in the nanomolar range (IC_50_ of 0.25–1 μg mL^−1^). Compared to azoles, there is a low frequency of *C. albicans* strains that are resistant to AmB [2]. The poor aqueous solubility of AmB (BCS Class IV) makes it difficult to solubilise in an adequate concentration in the small volume of saliva that is available in the oral cavity (1 mL) [3]. AmB is only commercialised as lyophilised formulations (micellar or lipidic nanoparticulate formulations) that are stable after reconstitution in aqueous media for a duration shorter than 24 h even when refrigerated [4,5] resulting in wastage, while poor patient compliance is an issue as formulations are not palatable. Taste masked orodispersable films (ODFs) can be potential solid dosage forms to deliver AmB in safe and efficient systems for the treatment of oropharyngeal fungal infections. Compared to other solid formulations, such as tablets and capsules, ODFs benefit from better patient compliance due to the ease of administration to dysphagic, paediatric and geriatric patients, without the need for water, and a rapid onset of action [6,7]. ODFs have shown better performance than semisolid formulations such as gels, because they can be easily transported, allow for accurate dosing and possess superior chemical and physical stability when packed appropriately, which can be important for unstable drugs in aqueous media such as AmB [8,9]. From an industrial and clinical point of view, ODFs would be more cost-effective formulations compared to parenteral AmB formulations, as they do not require sterilisation and lyophilisation, avoiding wastage. 

The hypothesis underpinning this work is that AmB-loaded ODFs prepared using GRAS (Generally Regarded as Safe) excipients would enhance the current therapies available for the treatment of fungal infections in the oral and pharyngeal cavities. However, to the best of our knowledge, this has not been achieved to date due to the physicochemical challenges when formulating this drug. Here, we present an optimised fast disintegrating ODF of AmB with improved stability, loaded with high amounts of drug and designed to ensure AmB solubility in small volumes of saliva, while being taste masked and locally effective. To ensure AmB solubility at the physiological pH of the oropharyngeal cavity, we entrapped AmB in sodium deoxycholate micelles that were then embedded within the ODF. Sodium deoxycholate was selected as it interacts with AmB forming micelles, while it accumulates in buccal tissue after penetration without causing a loss of superficial cell layers and interacts with the intercellular or membrane lipids increasing the permeability of drugs through the epithelium [10]. Design of experiment (DoE) studies enabled us to identify the optimal drug:excipient ratio needed to ensure high drug loading (1% AmB) that is critical in achieving local concentrations well above the IC_50_ against *Candida albicans*. The optimised ODF were fully characterised and their *in vitro* antifungal activity evaluated. 

## 2. Materials and Methods 

### 2.1. Materials

AmB was purchased from Azelis (Barcelona, Spain). Hydroxypropylmethyl cellulose acetate succinate (HPMC AS 912, Affinisol™), maltodextrin (Glucidex 12D) and sorbitol were a gift from DowPharma (Dewsbury, UK) and Roquette (Valencia, Spain). Microcrystalline cellulose (Avicel 200) and microcrystalline cellulose and sodium carboxymethylcellulose (Avicel CL-611) were kindly donated by FMC (Cork, Ireland), while hydroxypropyl cellulose (HPC, Klucel HXF) was a gift from Ashland (Barcelona, Spain). Dextrose and dextran from Leuconostoc mesenteroides (16 KDa) were purchased from Sigma (Madrid, Spain). Humidity capsules and stability chambers were purchased from Amebis Limited (Dunshaughlin, Ireland). All other chemicals and solvents were at least of ACS reagent grade and were used without further purification. 

### 2.2. Quality by Design (QbD) Optimisation of ODF 

Several critical quality attributes (CQAs) such as disintegration time in artificial saliva and the physical characteristics of the ODF (burst strength, flexibility, tackiness) were identified as key factors in order to meet the Target Product Profile (TPP) (Tables S1 and S2 in Supplementary Materials). A Taguchi design (L8 = 2^7) was carried out using Design Expert software 8.04 (Stat-Ease, Minneapolis, MN, USA). Seven formulation variables (factors) and two levels of each factor affecting the film formation were investigated (Table 1). Disintegration time, burst strength and appearance were evaluated as responses (Table 2).

#### 2.2.1. ODF Manufacture and Response Evaluation

Eight formulations of AmB-loaded ODFs (3 g each) (Table 2) were prepared as follows: dextrose-derived-polymer film former, Avicel 200 or Avicel CL-611, taste masking agent (5%) and plasticisers were weighed and mixed in a mortar and pestle. To this mixture, 3 mL of freshly prepared AmB-loaded micelles (30 mg of AmB and 24.6 mg of sodium deoxycholate [11]) was added and manually mixed until a homogenous mixture was formed. The cellulose-derived film formers were then added, if required, and mixed. Methanol was added to reduce the viscosity of the mixture to a pourable homogenous suspension that was immediately cast onto a release liner (Primeliner 36 µm 1S, Loparex BV, Apeldoorn, The Netherlands) using a coating knife (Multicator 411, Erichsen, Hemer, Germany) and film applicator (Erichsen Coatmaster 510 film applicator, Erichsen, Hemer, Germany), at a speed of 10 mm s^−1^ under vacuum (air pressure 60 Pa), to form a wet film with a thickness of 1000 μm. The film was allowed to dry under vacuum for 4–5 h. Once the films were dried, they were carefully removed from the release liner and properties of the film were evaluated (Table 2). 

Disintegration times of ODFs (1 × 1 cm) were measured in 3 mL of artificial saliva prepared as previously described: 14.4 mM sodium chloride, 16.1 mM mg potassium chloride, 1.31 mM calcium chloride dihydrate, 0.54 mM magnesium chloride hexahydrate, 1.96 mM dibasic potassium phosphate adjusted to pH 5.7 ± 0.01 [12] under gently shaking (30 slow 90° inversions of the vial per min). The mechanical properties (burst strength) of ODFs were evaluated using a texture analyser (Texture Analyser TA-XTplus, Stable Microsystems, Godalming, UK) attached to a film support rig (HDP/FSR, Stable Microsystems) [13]. For burst strength, the force required to rupture or break films was measured using a 5 mm spherical stainless-steel ball probe with probe adapter which was connected to the load cell. A film (35 × 15 mm) was placed in a film supporting rig and the moving probe reached the surface of the film with a pre-test speed of 2 mm s^−1^, test speed of 1 mm s^−1^ and post-test speed of 10 mm s^−1^. The force applied had a trigger load of 4.9 N and the force maximum (mN), travel distance (mm) and area under the curve (mN mm) were measured. Finally, the overall appearance of the films was evaluated as the sum of folding endurance, adhesion of the dried film to the release liner and homogeneity. Folding endurance was manually measured by counting the number of times the film could be folded at a 180° angle to the plane without breaking. A value of 1 was assigned for films with good/flexibility able to fold 180 degrees without breaking above 50 folds, 2 as medium flexibility films (25–50 times) and 3 as poor flexibility films, which break easily when folded more than 25 times. Adhesion of the dried film to the release liner was also taken into account, assigning a number from 1 to 3, with 1 indicating intact removal of the film and 3 complete breakage of the film upon removal. The homogeneity was also visually inspected, assigning 1 for films that exhibit a smooth surface with no cracks, 2 for films with some irregular surfaces and 3 for non-homogeneous films with lumps and/or cracks on the surface. 

#### 2.2.2. ODF Optimisation

Mathematical modelling was carried out by multiple linear regression analysis (MLRA). Only the statistically significant coefficients (*p* < 0.05) were considered in framing the polynomial equations, and the model was evaluated by analysing the *p*-value, coefficient of correlation (R^2^) and predicted residual sum of squares (PRESS) [14]. Films were optimised in order to minimise the disintegration time and improve overall appearance and mechanical strength. 

### 2.3. Full Physicochemical Characterisation of Optimised ODF

A full evaluation was performed including particle size and zeta potential (after disintegration in artificial saliva), powder X-ray Diffraction (PXRD), Fourier Transformed Infrared (FT-IR), Dynamic Vapour Sorption (DVS), Scanning Electron Microscopy (SEM) and surface area [11,14,15]. Briefly, particle size and zeta potential were measured in a Zetatrac after dilution (1 to 100) with artificial saliva. XRD measurements (*n* = 3) from 5° to 40° (2 theta) and a step scan rate 0.05° per second were performed in a Miniflex II Rigaku diffractometer with Ni-filtered Cu Kα radiation (1.54 Å) using a tube voltage and tube current of 30 kV and 25 mA respectively. FT-IR spectra were scanned in the range of 650–4000 cm^−1^ with a resolution of 4 cm^−1^ on a PerkinElmer Spectrum 1 FT-IR Spectrometer equipped with a UATR and a diamond/ZnSe crystal accessory. Baseline correction and data normalization were performed using Spekwin32 version 1.71.6.1. Water sorption kinetic profiles were obtained using a DVS (Advantage, Surface Measurement Systems, Alperton, UK) at 25.0 ± 0.1 °C. Samples (10–20 mg) were dried at 0% relative humidity (RH) for 1 h followed by step changes of 10% RH up to 90% RH, and the reverse for desorption. SEM was carried out in a Zeiss Supra Variable Pressure Field Emission Scanning Electron Microscope (Oberkochen, Germany) equipped with a secondary electron detector at 15 kV. Surface area was determined by the Brunauer, Emmett, Teller (BET) isotherm method using N2 adsorption with 6 points in the relative pressure range of 0.05–0.3 in a Micromeritics Gemini VI surface area analyser (Particular Sciences Ltd., Dublin, Ireland).

### 2.4. Content Uniformity 

ODF (1 × 1 cm) were weighed and disintegrated in 3 mL of deionised water prior to being diluted (1 to 2) with methanol to ensure full drug solubilisation. Samples were centrifuged to precipitate undissolved excipients (5000 rpm for 10 min) and AmB dissolved in the supernatant was quantified using a validated HPLC method [16]. Experiments were performed using films from ten different sections of the cast ODF (15 × 10 cm).

### 2.5. Release Studies in Artificial Saliva

ODF (1 × 1 cm) were dissolved in 10 mL of artificial saliva at 37 °C under slow magnetic stirring (50 rpm). Samples (1 mL) were obtained at 1, 2, 4, 6, 8, 10, 15 and 30 min and media were replaced with fresh artificial saliva. Withdrawn samples (0.1 mL) were mixed with methanol (0.1 mL), vortex and centrifuged (5000 rpm, 10 min) for HPLC analysis to determine the percentage of drug released. The remaining sample volume was centrifuged (500 rpm, 1 min) to precipitate undissolved excipients such as microcrystalline cellulose. The particle size of the supernatant was measured using a Malvern Zetasizer (Malvern Nano Zs, Malvern Instruments, Malvern, UK). Additionally, the supernatant was analysed by UV (300–450 nm, Multiskan GO, Thermo Scientific, Basingstoke, UK) to determine the aggregation state of the AmB [17]. The ratio of the absorbance at 332 nm corresponding to dimeric AmB versus the absorbance at 408 nm that corresponds to the monomeric state was plotted to demonstrate the prevalence of the dimeric aggregation state of AmB. 

### 2.6. In Vitro Antifungal Assays

In vitro antifungal activity was tested based on the agar diffusion assay as described by Ruiz et al. [1] according to the National Committee for Clinical Laboratory Standards (NCCLS) Method for antifungal disk diffusion susceptibility testing of yeast, standard M44-A2 [18]. In vitro activity was tested on three different *Candida* spp. (*C. albicans* CECT 1394, *C. parapsilosis* 57744 and *C. krusei* 52009 which was kindly provided by Dr. Pérez (CAQYM, University of Alcala de Henares, Alcalá de Henares, Spain) [1]. Strains were cultured in Sabouraud dextrose agar for 72 h to ensure viability and absence of contamination at 35 °C (±2 °C). Antifungal tests were carried out in Müeller Hinton agar (MHA) supplemented with glucose (2% *w/v*) and methylene blue (0.5 mg/mL). Inoculum was prepared by picking a few distinct colonies, which were suspended in 3 mL of sterile saline (0.9%). The resulting suspension was vortexed and its turbidity was adjusted with a spectrophotometer by adding sufficient sterile saline or more colonies to adjust the transmittance to that produced by a 0.5 McFarland standard at 530 nm wavelength, resulting in a yeast stock suspension of 1 × 10^6^ cells per mL. Yeast suspension was inoculated to the MHA (200 mL) and was casted in disposable sterile petri dishes (instead of spreading it on the surface of the plate as specified in M44-A2). Once solidified, AmB ODFs (circles with a 6 mm diameter) were tested. Four disks were placed in each plate. AmB ODF in vitro activity was compared to commercially available AmB Neo-Sensitabs tablets (10 µg, 6 mm tablets from Rosco diagnostic A/S, Taastrup, Denmark) and AmB impregnated on inoculation 6 mm paper disks (10 µg/20 µL of DMSO) with appropriate DMSO controls. Once the disks were placed on the surface of the agar, the plates were inverted and placed in an incubator set to 35 °C (±2 °C) within 15 min after the disks were applied. After 24 h of incubation, the inhibition halo was measured. 

### 2.7. Stability Studies

Physicochemical stability studies were performed under accelerated conditions (40, 60 and 80 °C) for one week and long term at 5 ± 3 °C and 25 ± 3 °C. AmB ODF (1 × 1 cm) were placed in sealed vials into Amebis chambers (Amebis Ltd., Dublin, Ireland) at the selected temperature. A sensor cap was used to seal the test chamber and a logger cap connected to the sensor cap was used to collect and transmit the temperature and humidity test conditions wirelessly to the Amebis Control Software [14]. Disintegration time and drug content were quantified at different time points (time zero, day 1, 3 and 7). The degradation rate of AmB was calculated by fitting the percentage of drug degraded at different time points to several degradation kinetic equations (zero order, first order, second order, Avrami and diffusion) and the best fitted degradation kinetic model was selected (i.e., highest R^2^). Using the degradation rates at different temperatures, the Arrhenius equation was employed to calculate the activation energy (Equation (1)):

(1) $$K=A\;e^{\frac{-Ea}{RT}}$$

where *K* is the degradation rate (% drug degraded/day), *A* is the collision factor, *T* is the absolute temperature in Kelvin, *R* is the gas constant (1.985 cal/mol/K) and *Ea* is the activation energy in cal/mol [14]. Drug stability at room temperature was then predicted using the Arrhenius equation and compared to experimental values.

## 3. Results

### 3.1. QbD Studies for Optimisation of AmB-Loaded ODFs

**Figure 1:**
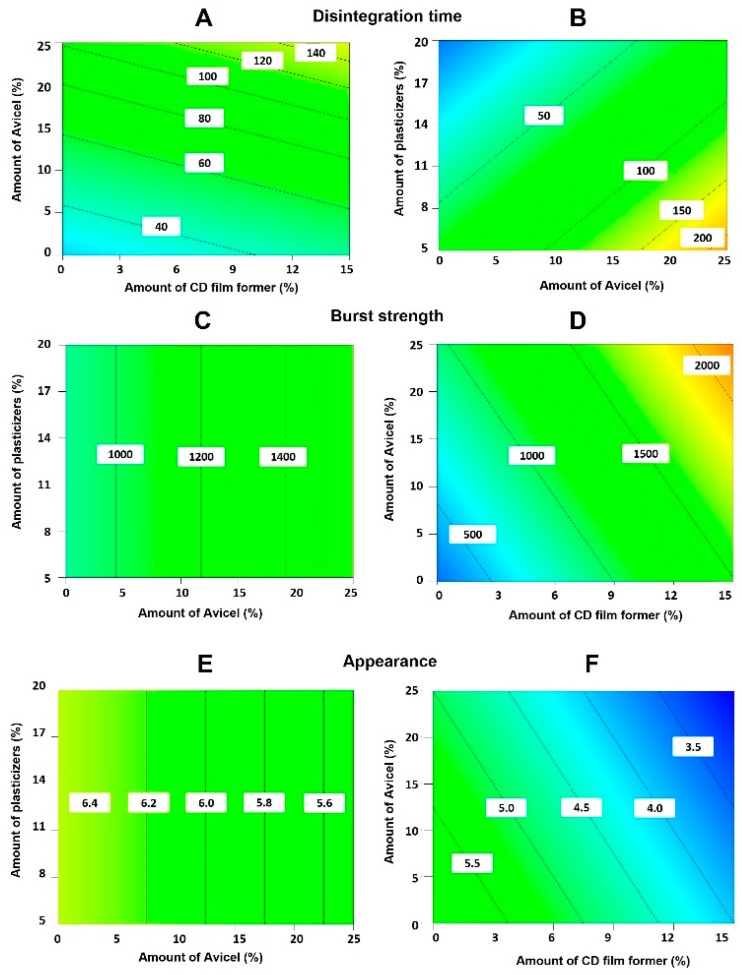
Contour plots showing the influence of the most influential factors affecting the disintegration time (**A**,**B**), the burst strength (**C**,**D**) and the overall appearance (**E**,**F**) of the AmB-loaded orodispersable films (ODFs). Key: CD, cellulose-derived.

The first-order mathematical model generated for each response variable was found to be statistically significant (*p* < 0.05 in each case). Co-efficients with *p* values > 0.1 were considered insignificant based on Pareto charts and ANOVA analysis. High R^2^ values for the polynomial equations obtained for all the response variables indicate a good fit to experimental data (Table S3). The variables, type and amount of Avicel and number of plasticizers, showed a significant effect on the disintegration time of the ODFs (Figures S1 and S2). ODFs with lower disintegration time were obtained when lower amounts of Avicel and cellulose-derived film formers were used. The disintegration time was reduced when higher percentages of plasticisers were employed and when Avicel 200 was incorporated in the film compared to Avicel CL-611, as the latter acts as a viscosity enhancing agent resulting in thixotropic gels, which retard disintegration (Figure 1A,B). 

The choice of dextrose-derived film former, volume of methanol added, type/amount of Avicel and amount of cellulose-derived film formers had a significant impact on burst strength (Figures S3 and S4). The use of maltodextrin and Avicel 200 resulted in films with higher burst strength. The higher the amount of Avicel, HPMC AS/HPC and methanol, the better the mechanical strength of the films (Figure 1C,D). 

The appearance of the films was rated as described above (Figure S5). DoE indicated that the main variables that contributed to the appearance of the film were: the type of dextrose-derived film former and the amount of cellulose-derived film formers utilised, followed by type and amount of Avicel and taste masking agent (Figures S6 and S7), although results were not statistically significantly different. The appearance of the film was smoother when maltodextrin was used, although high amounts of maltodextrin increased film tackiness. When higher amounts of Avicel and cellulose-derived film formers were used, appearance was improved. Avicel 200 and sorbitol resulted in smoother film surfaces compared to dextrose films as dextrose can recrystallize resulting in rougher surfaces [19] (Figure 1E,F).

### 3.2. Manufacturing of Optimised AmB ODF

A trade-off between key CQAs was necessary to attain optimal characteristics, i.e., short disintegration time (which is critical for faster onset of action), maximal burst strength (in order to obtain robust films that can be easily manufactured and packaged without breaking), and good appearance. A closer match to ideal CQAs was obtained with sorbitol, Avicel 200 (10%), 20% of plasticisers (PEG 400:glycerol, 1:1 *w/w*) and 10% of cellulose-derived film formers (HMPC 912 AS:HPC, 1:4 weight ratio). Regarding the type of dextrose-derived film former, maltodextrin conferred better flexibility and faster release from the films compared to those obtained with dextran, as dextran interacts more strongly compared to microcrystalline cellulose or the modified cellulose-derived film formers. However, high amounts of maltodextrin significantly increased adhesion of the films to the release liners. Thus, we decided to optimise the ODF using a mixture of dextran and maltrodextrin (1:1 *w/w*) (Table 3). The films exhibited a good overall appearance with a dried thickness of 0.14 ± 0.01 mm, a weight of 28.5 ± 1.5 mg/cm^2^, a drug content of 0.996 ± 0.045 mg/g (which was uniform with a low standard deviation and a variance coefficient of 4.5%), a burst strength of 2190 mN mm and a disintegration time of ≤60 s in 3 mL of artificial saliva. 

**Figure 2:**
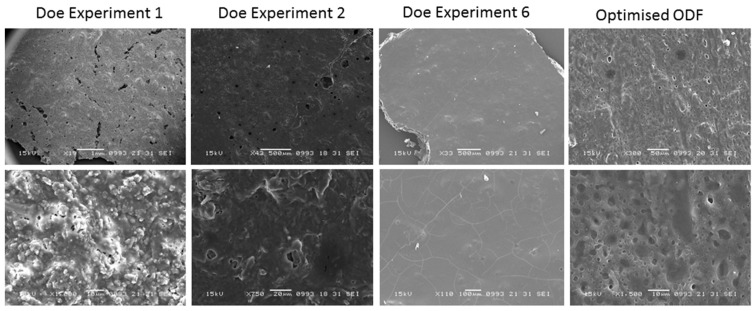
Scanning Electron Microscopy (SEM) micrographs of optimised AmB-loaded ODFs compared to those obtained in the Taguchi DoE before optimization. Bars; DOE Experiment 1—Top: 1 mm and Bottom: 10 μm, DOE Experiment 2—Top: 500 μm and Bottom: 20 μm, DOE Experiment 6—Top: 500 μm and Bottom: 100 μm, Optimised ODF—Top: 50 μm and Bottom: 10 μm.

SEM micrographs revealed a smooth and porous surface of the optimised AmB-loaded ODF compared to films obtained in the DoE experiment 1 and 2, which exhibited a granular rough texture or embedded crystals respectively probably due to the use of dextran in the first film instead of maltodextrin and the presence of dextrose in DoE 2 ODF which tends to crystallise (Figure 2). Films obtained in DoE 6 experiments appeared cracked and exhibited low elasticity likely due to the low percentage of plasticisers and Avicel 200 included in the formulation. 

### 3.3. Performance and Further Characterisation of the Optimised AmB-Loaded ODF

**Figure 3:**
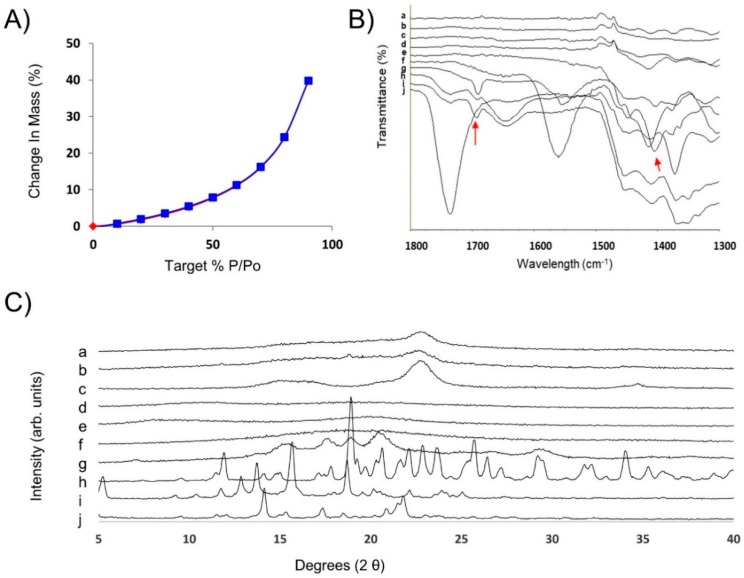
Physicochemical characterization of optimised AmB-loaded ODFs. (**A**) Water sorption kinetic profile. (**B**) FTIR spectra: (a) hydroxypropyl cellulose (HPC), (b) maltodextrin, (c) dextran, (d) Avicel 200, (e) sorbitol, (f) sodium deoxycholate, (g) AmB, (h) AmB-loaded ODF, (i) physical mixture, (j) HPMC 912 AS. (**C**) PXRD patterns: (a) AmB-loaded ODF, (b) Physical mixture of all components, (c) Avicel 200, (d) HPMC 912 AS, (e) HPC, (f) maltodextrin, (g) dextran, (h) sorbitol, (i) sodium deoxycholate, (j) AmB.

The smooth and porous surface observed by SEM can be associated with the high 2.3 ± 0.5 m^2^/g surface area of the optimized film (Figure 2). AmB-loaded ODFs showed a 40% increase in mass at 90% relative humidity, associated with a large water uptake due to the films hydrophilicity as shown by the water sorption kinetic profile (Figure 3A). No mass loss (associated to phase transformation/crystallization) was observed during the sorption or desorption cycle. The solid state of the film was retained (as demonstrated by post DVS XRD analysis (Figure S8) indicating an overall acceptable physical stability. The FTIR spectra showed a broadening of the peak at 1691 cm^−1^ (C=O stretch) probably attributed to hydrogen bonding between the AmB and excipients such as sodium deoxycholate, sorbitol and acetate succinate groups of the HPMC AS (Figure 3B and Figure S9). 

The XRD pattern of the physical mixture of all components revealed crystalline Bragg peaks attributed to sorbitol (Figure 3B(b,h)), which are not present in the casted film. However, a characteristic halo attributed to the semi-crystalline nature of the microcrystalline cellulose (Avicel 200) was observed in the optimised ODFs. Lower intensity values at several Bragg peaks (5°, 14.15°, 17.35°, 21.75°) were observed in the diffractogram of the AmB-loaded ODF compared to the physical mixture, which can be attributed to the presence of amorphous AmB-sodium deoxycholate complexes in the films. However, bearing in mind that the AmB content in the ODF is 1%, it is likely that the results obtained from XRD measurements are not conclusive due to the XRD detection limit for crystalline AmB.

### 3.4. Release and Aggregation State

**Figure 4:**
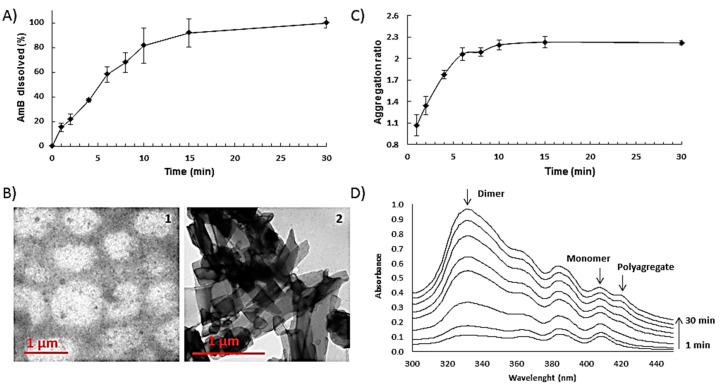
Release profile, morphology and aggregation state of optimised AmB loaded ODFs. Key: (**A**) Release profile of AmB-loaded ODFS in artificial saliva. (**B**) Transmission electron microscopy (TEM) of AmB-loaded ODF after reconstitution in aqueous media (left image) and crystalline unprocessed AmB (right image); (**C**) Aggregation ratio during the release studies; (**D**) Transformation of AmB aggregation states over time.

Aligned with the high porosity of the films, ODF presented a fast-dissolving behaviour (>80% in 10 min) in saliva (Figure 4A). Once disintegrated, particle size and zeta potential of the resulting suspension was measured. Initial particle size was bimodal mainly dominated (>70%) by large particles (>1 µm) due to insoluble excipients. After centrifugation, a white pellet and a transparent yellow supernatant was obtained. The latter demonstrated a bimodal particle size distribution of 8.7 ± 2.5 nm and 918 ± 120 nm with an anionic zeta potential of −14 ± 3 mV, indicating that after disintegration of the ODFs, AmB remained solubilised in the supernatant in equilibrium between micelles and particles close to 1 μm in size. TEM images confirmed the presence of micelles and particles of that size (Figure 4(B1)). Characteristic crystals corresponding to unprocessed AmB (Figure 4(B2)) were not observed after disintegration of the ODF and release of the AmB in the media which supports drug solubilisation within micelles. 

Regarding the aggregation state of AmB released from the films, UV spectroscopy indicated a shift from the monomeric to dimeric state illustrated by the faster increase in the peak at 332 nm compared to the one at 408 nm corresponding to the monomeric state (Figure 4C,D). At the earlier time points (1–2 min), there is an equilibrium in solution between monomer and dimer. As time progresses and a higher percentage of AmB is released to the media, AmB self-aggregates shifting from monomer to dimer after 2 min and from dimer to polyaggregate after 8 min (peak at 420 nm) [17,20]. AmB solubility can be enhanced by the presence of PEG 400 and sodium deoxycholate in the formulation [21]. Fungizone^®^, a commercial AmB parenteral formulation, consists of 1:2 AmB:sodium deoxycholate molar fraction that facilitates the solubilisation of AmB in dimeric form within micelles [20]. The presence of nanometric (≈10 nm) spherical single layer particles in solution after ODF disintegration can be explained by the partial solubilisation of the drug within sodium deoxycholate micelles. However, even though initially AmB is incorporated in the solid mixture of excipients solubilised in sodium deoxycholate, the later addition of solvent to produce a pourable castable formulation could destabilise initially formed AmB sodium deoxycholate micelles. Thus, the different aggregation states of released AmB can be a consequence of the initial solubilisation of AmB in the monomer state in the presence of PEG 400 and the formation of ion pairs with sodium deoxycholate, followed by the encapsulation of AmB within micelles resulting in prevalence of the dimeric state. When higher amounts of AmB are released over time in the media, AmB self-aggregates into polyaggregates close to 1 μm in size which are stabilised by the release of dextrose-based polymers, in particular maltodextrin, which has been previously shown to be able to effectively stabilise dispersed systems such as oil-in-water emulsions [22].

### 3.5. Antifungal Activity

**Figure 5:**
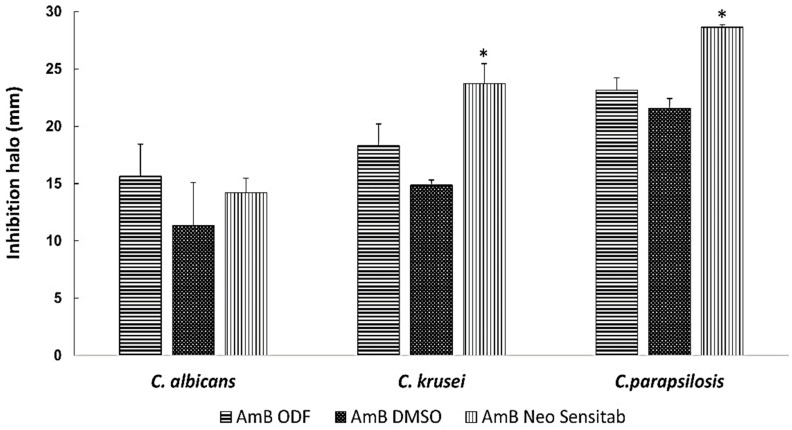
In vitro activity against *C. albicans*, *C. krusei* and *C. parapsilopsis*. The isolates were classified as susceptible (S) to AmB when the inhibition zone was ≥15 mm, resistant (R) when it was ≤10 mm and intermediate (I) or susceptible-dose dependent when the inhibition zone was between 10- and 15-mm. Inhibition zone diameters are expressed as mean ± SD in mm. All experiments were performed in triplicate. * *p* < 0.05 One-way ANOVA test.

The optimised AmB-loaded ODFs showed good in vitro antifungal activity against the three *Candida* spp. (with an inhibition zone >15 mm) equivalent to that of AmB dissolved in DMSO and the commercially available disks, which indicates good drug release from the film and diffusion across the agar of the solubilised AmB (Figure 5). Bearing in mind the potency of the drug against *C. albicans, C. parapsilopsis* and *C. krusei* (MIC_50_ ranges from 0.25–1 µg/mL) reported in the literature [2], the dose delivered by a 1 × 1 cm film would be adequate to ensure efficacy against buccal candidiasis. Considering that the drug loading is 1% *w/w* and that a 1 cm^2^ film weights around 30 mg, each 1 cm^2^ film contains approximately 0.3 mg of AmB. Assuming a volume of ~1 mL in the oral cavity [3,23], the concentration of AmB would be 300 µg/mL, which is well above the MIC_50_ reported in literature. Even if the concentration is diluted further with 10 mL or 100 mL i.e., 10 or 100-fold, the AmB concentration would be in range between 3 or 30 µg/mL, which are concentrations still above the MIC_50_ and, for this reason, we believe that the drug levels would be adequate to elicit a pharmacological effect.

### 3.6. Physicochemical Stability

**Figure 6:**
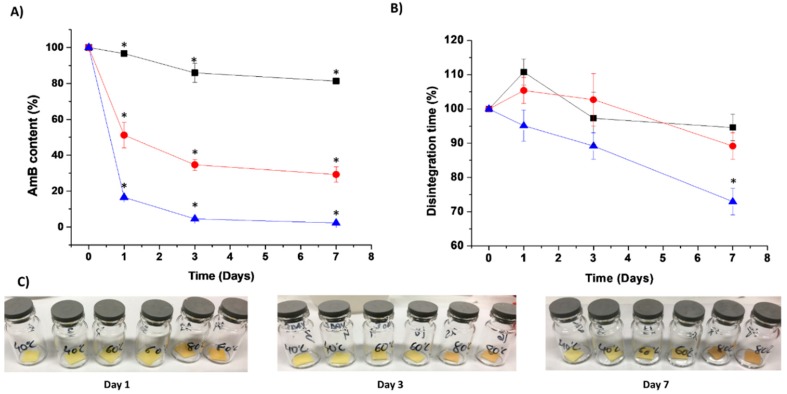
Physicochemical stability studies of optimised AmB-loaded ODFs at different temperatures. Key: -■- 40 degrees, -●- 60 degrees, -▲- 80 degrees. (**A**) Chemical stability expressed as AmB content. (**B**) Change in disintegration time for films stored at different temperatures. (**C**) Physical appearance. * *p* < 0.05 indicative of statistically significant difference compared to AmB content and disintegration time of films at time 0 (One-way ANOVA with a post-hoc Tukey’s test, level of significance set at 5%).

Stability studies showed that the optimised AmB-loaded ODF was physiochemically stable over a year (>90% drug content) at 5 °C under desiccated conditions. Accelerated stability studies demonstrated a pronounced chemical degradation (80 °C >> 60 °C >> 40 °C), while a change in colour occurred at the highest temperature (Figure 6). The disintegration times of ODFs subject to accelerated stability studies was reduced over time, probably due to the evaporation of bound water within the film and the formation of micropores (Figure 6). However, in all the tested conditions, the disintegration time remained below 2 min as considered appropriate for fast-disintegrating ODFs. The Avrami kinetic model fitted the degradation of AmB from loaded ODFs. This kinetic model is commonly applied to evaluate the growth and formation of crystals [24]. Nevertheless, several authors have also used this model in the stability prediction of nanocomposites [25,26]. The breaking of a 3D network becomes more heterogeneous as the degradation progresses, and thus, the degradation proceeds faster as the combined effects of physical and chemical degradation occur simultaneously, especially at higher temperatures [26]. The activation energy of the AmB-loaded ODFs was 5.71 Kcal/mol, which correlates with the poor physicochemical drug stability observed at high temperatures (Figure 6). Experimental and predicted data from the Arrhenius equation showed that ODFs stored at 25 °C at desiccated conditions remained stable over 15 days (>90% drug content, disintegration time of 55 s). Thus, our AmB ODFs would remain stable for 2 weeks at room temperature in a closed pouch, which is a significant advantage to existing AmB commercialised parenteral formulations that are unstable after 24 h of reconstitution of the lyophilised powder [4] reducing overall cost of treatment and wastage.

## 4. Discussion

To the best of our knowledge, this is the first report of AmB, a high molecular weight poorly water-soluble antifungal, being formulated as an ODF for the treatment of oropharyngeal candidiasis. The optimised ODF was achieved by combining dextran and maltodrextrin as dextrose-derived polymer film formers with sorbitol for taste masking, microcrystalline cellulose (Avicel 200) for enhancing mechanical strength, PEG 400 and glycerol as plasticizers to ensure faster disintegration and adequate plasticity and HMPC 912 AS /HPC for facilitating film formation. The combination of PEG 400 and glycerol (1:1 weight ratio) was selected based on previous results (data not shown). Glycerol improves mouthfeel, which is a necessity in an ODF formulation, as well as enhancing the viscosity resulting in higher, unacceptable disintegration times. In contrast, PEG 400 decreases disintegration time of ODF and, in combination with HPMC, gives optimal films in terms of tensile strength and flexibility [27]. 

Currently, nystatin (which has a similar chemical structure to AmB) formulated as a suspension is considered as the reference treatment for oral candidiasis; nevertheless, a recent meta-analysis demonstrated a limited efficacy for nystatin, lower than fluconazole, in treating oral candidiasis in infants, children and HIV patients, which is related to a poor and variable bioavailability in the oral mucosa [28]. AmB has shown 4-fold higher in vitro efficacy than nystatin [29], but its poor aqueous solubility limits its use in clinical practice, and it is only marketed as intravenous formulations. After disintegration of the ODF, AmB is maintained solubilised in the aqueous media in equilibrium between different aggregation states mainly monomer and dimer at earlier times points, which have shown to possess much greater efficacy than polyaggregates against *Candida* [30,31]. Unlike previous studies in which the antifungal activity was tested after disintegration of the films in liquid form [32], enhanced in vitro activity of the AmB-loaded ODF was shown here for the intact film placed on top of the agar, showing that the release and diffusion of the drug across the agar occurs and hence AmB is free to elicit its effect. Excipients utilised in ODFs are used within ranges that are considered GRAS (generally regarded as safe). Sodium deoxycholate, at the concentrations used in optimised the ODFs, has been shown to cause no major morphological changes [10]. Although further toxicological and in vivo efficacy studies are required, we have shown that this technology platform can be a promising AmB formulation for the treatment of oral candidiasis.

## 5. Conclusions

Fast-dissolving orodispersible films containing a very poorly soluble and unstable drug, AmB, have been successfully engineered and prepared using a solvent casting film method in which the drug is solubilised at a final concentration of 1% *w/w* upon disintegration of the film. The optimised AmB orodispersible flms consisted of a mixture of dextrose-derived polymers (25% dextran and 25% maltodextrin) and cellulose-derived film formers (3% HMPC AS and 12% HPC) with 10% Avicel 200 added to enhance the mechanical strength of the film, 5% sorbitol for taste masking, and 10% PEG 400 and 10% glycerol as plasticizers. The optimised ODF exhibited a fast disintegration time (60 ± 3 s), quick release in artificial saliva (>80% in 10 min), high burst strength (2190 mN mm), good chemical stability (1 year at refrigerated conditions and 2 weeks at room temperature) and high efficacy against several *Candida* spp. (*C. albicans, C. parapsilosis* and *C. krusei* with an inhibition halo >15 mm). Although further toxicological and in vivo efficacy studies are required, these novel orodispersible films prepared with GRAS excipients have potential in the treatment of oro-and pharyngeal candidiasis, and hence represent a promising system with wide applications in clinical practice among immunocompromised patients suffering from this disease. 

## Figures and Tables

**Table 1:** Formulation and process variables with their respective high and low levels included in the Taguchi screening design.

Variables	Levels	
	Low (−1)	High (+1)
Type of dextrose-derived-polymer film former	Maltodextrin	Dextran
Taste masking agent (5%)	Sorbitol	Dextrose
Type of binder/suspending agent	Avicel 200	Avicel CL-611
Amount of Avicel polymers (%)	0	25
Amount of total plasticisers (PEG 400: glycerol, 1:1 *w/w*) (%)	5	20
Methanol	Low (1–2 mL)	High (>3 mL)
Amount of cellulose-derived film formers (HPMC 912 and HPC in a 1:4 weight ratio) (%)	0	15

**Table 2:** Design matrix of AmB-loaded orodispersable films (ODFs) prepared as per the Taguchi design. The overall appearance of the film was evaluated as the sum of the folding endurance, adhesion and homogeneity. Regarding the folding endurance, a value of 1 was assigned for films with good/flexibility able to fold 180 degrees without breaking above 50 folds, 2 as medium flexibility films (25–50 times) and 3 as poor flexibility films, which break easily when folded more than 25 times. Adhesion of the dried film to the release liner was also taken into account, assigning a number from 1 to 3—with 1 allowing intact removal of the film and 3 complete breakage of the film upon removal. The homogeneity was ranked taking into consideration the value of 1 for films that exhibit a smooth surface with no cracks, 2 for films with some irregular surfaces and 3 for non-homogeneous films with lumps and/or cracks on the surface.

Run	Type of Dextrose-Derived-Polymer Film Former	Taste Masking Excipient	Type of Avicel	Amount of Avicel (%)	Amount of Total Plasticisers (%)	Amount of Methanol	Amount of Cellulose-Derived Film Formers (%)	Disintegration Time (Seconds)	Flexibi- lity	Adhesion	Appea-rance	Burst Strength (Mn mm)
**1**	Dextran	Sorbitol	200	25	5	1	15	270	2	1	4	1300.9
**2**	Maltodextrin	Dextrose	200	25	20	2	15	90	1	1	3	2468
**3**	Maltodextrin	Dextrose	200	0	5	1	0	60	2	3	6	0
**4**	Dextran	Dextrose	CL611	25	5	2	0	360	3	2	8	0
**5**	Maltodextrin	Sorbitol	CL611	25	20	1	0	165	2	3	6	0
**6**	Maltodextrin	Sorbitol	CL611	0	5	2	15	150	2	1	4	1061
**7**	Dextran	Sorbitol	200	0	20	2	0	15	3	1	7	0
**8**	Dextran	Dextrose	CL611	0	5	1	15	240	3	2	8	0

**Table 3:** Composition and properties of the optimised AmB-loaded ODFs.

**Composition**	
Dextran	25% (0.74 g)
Maltodextrin	25% (0.74 g)
Sorbitol	5% (0.15 g)
Avicel 200	10% (0.3 g)
Plasticisers (PEG 400: Glycerol, 1:1 *w/w*)	20% (0.3 g + 0.3 g)
Simil Fungizone (9)	3 mL (=30 mg AmB + 24.6 mg sodium deoxycholate)
Cellulose-derived film formers	15% (0.09 g HMPC AS + 0.36 g HPC)
Methanol	1.5 mL
**Properties**	
Disintegration time	60 ± 3 s
Burst strength	2190 ± 140 mN mm
Overall score of appearance	4 = Flexibility (1), tackiness (2) and homogeneity (1)

## Supplementary Materials

The following are available online at https://www.mdpi.com/1999-4923/11/8/369/s1, Figure S1: Pareto charts depicting the effect of (C) Type of Avicel, (D) Amount of Avicel, (E) Amount of plasticisers and (G) Amount of cellulose-derived film formers on the disintegration time. Orange colour indicates a positive effect whereas blue colour indicates a negative effect. Figure S2: Effect of the four variables (Type of Avicel, amount of Avicel, amount of plasticizers and amount of cellulose-derived film formers) on the disintegration time. Figure S3: Pareto charts depicting the effect of (A) Type of dextrose-derived film former, (C) Type of Avicel, (D) Amount of Avicel, (F) Volume of methanol and (G) Amount of cellulose-derived film formers on the burst strength of the film (expressed as AUC). Orange colour indicates a positive effect whereas blue colour indicates a negative effect. Figure S4: Effect of the significant variables (Film former, Type of Avicel, amount of Avicel and amount of cellulose-derived film formers) on the burst strength expressed as AUC of the film. Figure S5: Appearance of the eight AmB-loaded films prepared according to Taguchi matrix design. Figure S6: Pareto charts depicting the effect of (A) Type of dextrose-derived film former, (B) Taste masking, (C) Type of Avicel, (D) Amount of Avicel and (G) Amount of cellulose-derived film formers on the appearance of the film. Orange colour indicates a positive effect whereas blue colour indicates a negative effect. Figure S7: Effect of the five variables with higher impact on the final appearance of the film (Type of Avicel, amount of Avicel, taste masking agent, type of dextrose-derived film former and amount of cellulose derived-film formers). Figure S8: PXRD patterns of raw materials and AmB-loaded ODF before and after DVS analyses. Key: AmB ODF post DVS, (b) AmB ODF, (c) physical mixture, (d) Avicel 200, (e) HPMC AS; (f) HPC, (g) maltodextrin, (h) dextran, (i) sorbitol, (j) sodium deoxycholate, (k) AmB. Figure S9: FT-IR spectra. (a) HPC, (b) maltodextrin, (c) dextran, (d) Avicel 200, (e) sodium deoxycholate, (f) sorbitol, (g) HPMC 912 AS, (h) AmB, (i) physical mixture, (j) AmB-loaded. Table S1: Target product profile (TPP) elements for AmB-loaded ODFs. Table S2: Critical quality attributes (CQAs) of AmB-loaded ODFs. Table S3: Co-efficient values and statistical parameters obtained for first order equations for the studied response variables: 1-Type of dextrose-derived-polymer film former, 2-Taste masking agent, 3-Type of Avicel, 4-Amount of Avicel, 5-Amount of plasticisers, 6-Amount of methanol, 7-Amount of cellulose-derived film formers. Results were analysed using a first order equation ($Y=B_{0}+B_{1}X_{1}+B_{2}X_{2}+B_{3}X_{3}+B_{4}X_{4}+B_{5}X_{5}+B_{6}X_{6}+B_{7}X_{7}$) generated for the response variables investigated in the DoE. Seven coefficients (B1 to B7) were calculated with B0 as the intercept. Only those coefficients which were significant were retained in the simplified equations.
