# Supplementary material for: Designing Fast-Dissolving Orodispersible Films of Amphotericin B for Oropharyngeal Candidiasis
Source: Pharmaceutics. 2019 Aug 1;11(8):369. doi: 10.3390/pharmaceutics11080369 (PMC6723921; doi:10.3390/pharmaceutics11080369)
Supplement: Supplementary file 1 [file pharmaceutics-11-00369-s001.pdf]

pharmaceutics

Supplementary Materials: Designing Fast-Dissolving Orodispersible Films of Amphotericin B for Oropharyngeal Candidiasis

Dolores R. Serrano, Raquel Fernandez-Garcia, Marta Mele, Anne Marie Healy and Aikaterini Lalatsa

Table 1. Target product profile (TPP) elements for AmB-loaded ODFs.

TPP Elements	Target	Justification
Dosage form	ODF	AmB-loaded ODF could enhance the efficacy against buccal candidiasis in immunocompromised patients, while reducing the toxicity compared to oral or parenteral formulations.
Route of administration	Oral /Buccal	Good patient compliance and better targeting.
Dosage strength	1 mg	Taking into account the volume of saliva, 1 mg would deliver a concentration above the IC_{50} against most fungal strains.
Stability	At least 12 months at room temperature	To maintain drug efficacy during storage period. Micelles incorporated within a film matrix enhance higher storage stability.

Table 2. Critical quality attributes (CQAs) of AmB-loaded ODFs.

CQA	Target	Is it a CQA?	Justification
Appearance	Visual appearance acceptable without cracks or lumps on the surface	Yes	Visual appearance critical for patient compliance.
Size	1 × 1 cm	Yes	Dose has to be contained within a maximum size of 1 × 1 cm films.
Taste	No unpleasant taste	Yes	Taste is critical in ODFs to ensure patient compliance.
Disintegration time	Below 1 min	Yes	Disintegration affects efficacy of the formulation.
Drug release	Fast	Yes	Fast onset of action needed for clinical efficacy and patient compliance
Physical characteristics	Adequate burst strength, flexibility and low tackiness	Yes	Ensures physical stability of the ODFs during packaging and administration.
Content uniformity	Meet Pharmacopeia requirements	Yes	Variability in content uniformity affects safety and efficacy.

Table 3. Co-efficient values and statistical parameters obtained for first order equations for the studied response variables: 1-Type of dextrose-derived-polymer film former, 2-Taste masking agent, 3- Type of Avicel, 4-Amount of Avicel, 5- Amount of plasticisers, 6-Amount of methanol, 7-Amount of cellulose-derived film formers.Results were analysed using a first order equation ($Y = B_0 + B_1X_1 + B_2X_2 + B_3X_3 + B_4X_4 + B_5X_5 + B_6X_6 + B_7X_7$) generated for the response variables investigated in the DoE. Seven coefficients (B1 to B7) were calculated with B0 as the intercept. Only those coefficients which were significant were retained in the simplified equations.

**Coefficient code**	**First-order polynomial coefficient for response variables**		
	**Disintegration time (seconds)**	**Burst strength (mN*mm)**	**Appearance**
B_0	+2.02	+604.75	+5.75
B_1 (term A)	-	−277.50	+1.0
B_2 (term B)	-	-	−0.5
B_3 (term C)	+0.19	−339.50	+0.75
B_4 (term D)	+0.27	+339.50	−0.5
B_5 (term E)	−0.24	-	-
B_6 (term F)	-	+277.50	-
B_7 (term G)	+0.093	+604.75	−1
R^2	0.973	0.999	0.96

Disintegration time = 2.02 +0.19 (Type of Avicel) + 0.27 (Amount of Avicel) -0.24 (Amount of plasticisers) +0.093 (Amount of cellulose-derived film formers).

Burst strength = +604.75 -277.5 (Type of dextrose-derived-polymer film former)-339.5 (Type of Avicel) +339.5(Amount ofAvicel) +277.5 9 (Volume of methanol) + 604.75 (Amount of cellulose-derived film formers).

Appearance= +575 +1 (Type of dextrose-derived-polymer film former) -0.5 (Taste masking) +0.75 (Type of Avicel) -0.5 (Amount of Avicel) -1(Amount of cellulose-derived film formers).

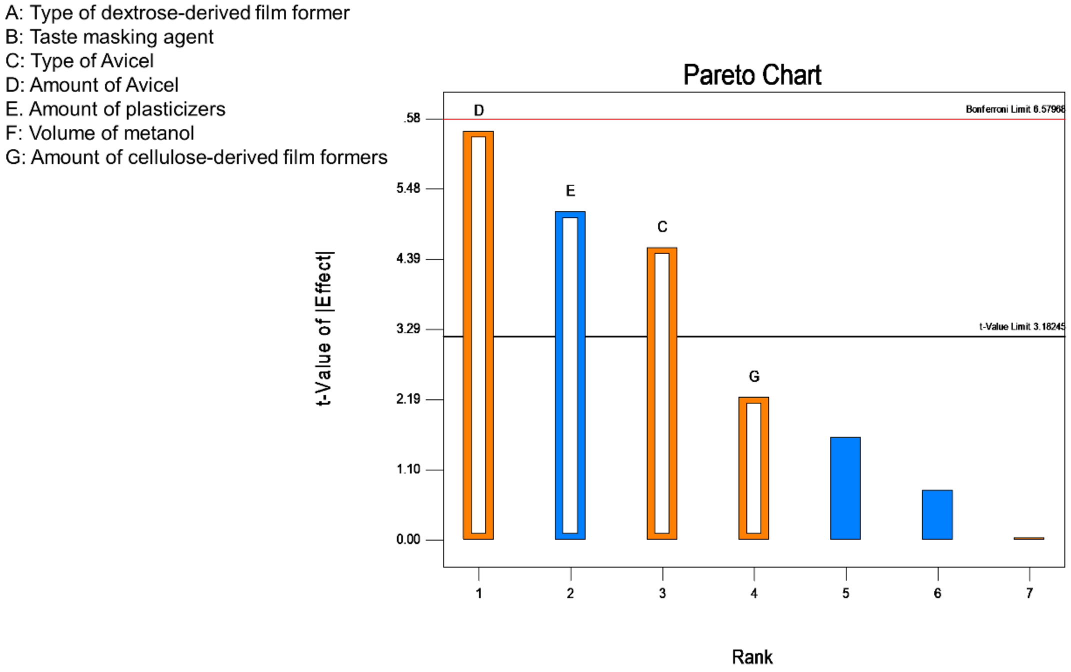

Figure 1. Pareto charts depicting the effect of (C) Type of Avicel, (D) Amount of Avicel, (E) Amount of plasticisers and (G) Amount of cellulose-derived film formers on the disintegration time. Orange colour indicates a positive effect whereas blue colour indicates a negative effect.

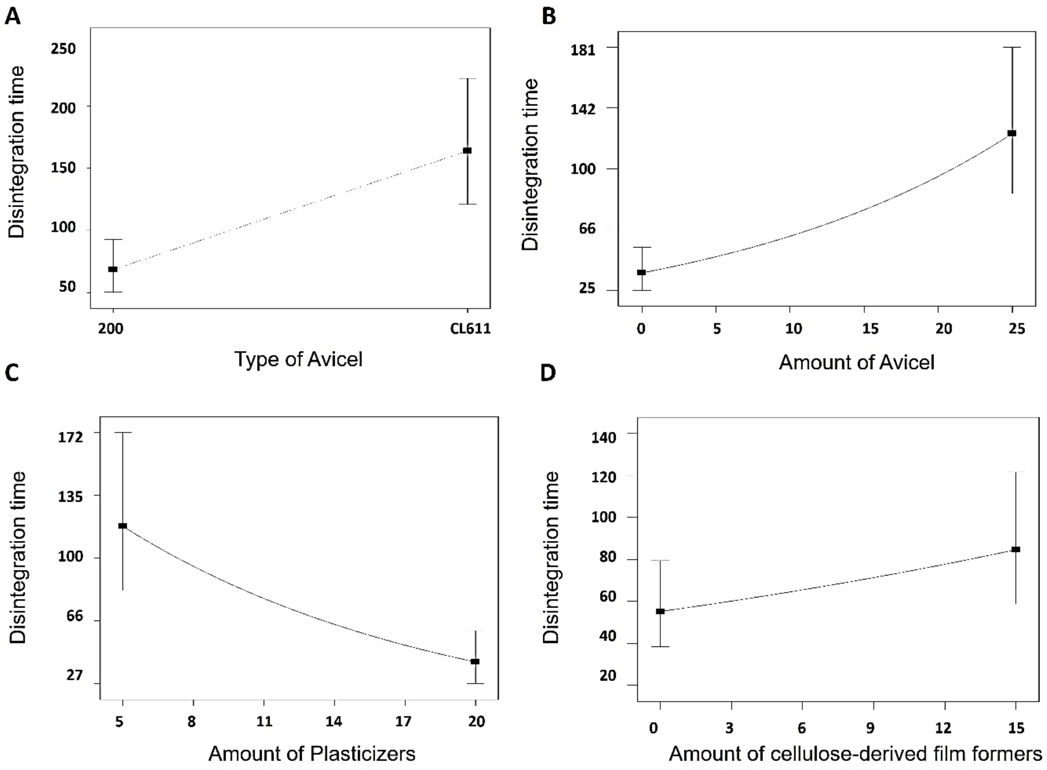

Figure 2. Effect of the four variables (Type of Avicel, amount of Avicel, amount of plasticizers and amount of cellulose-derived film formers) on the disintegration time.

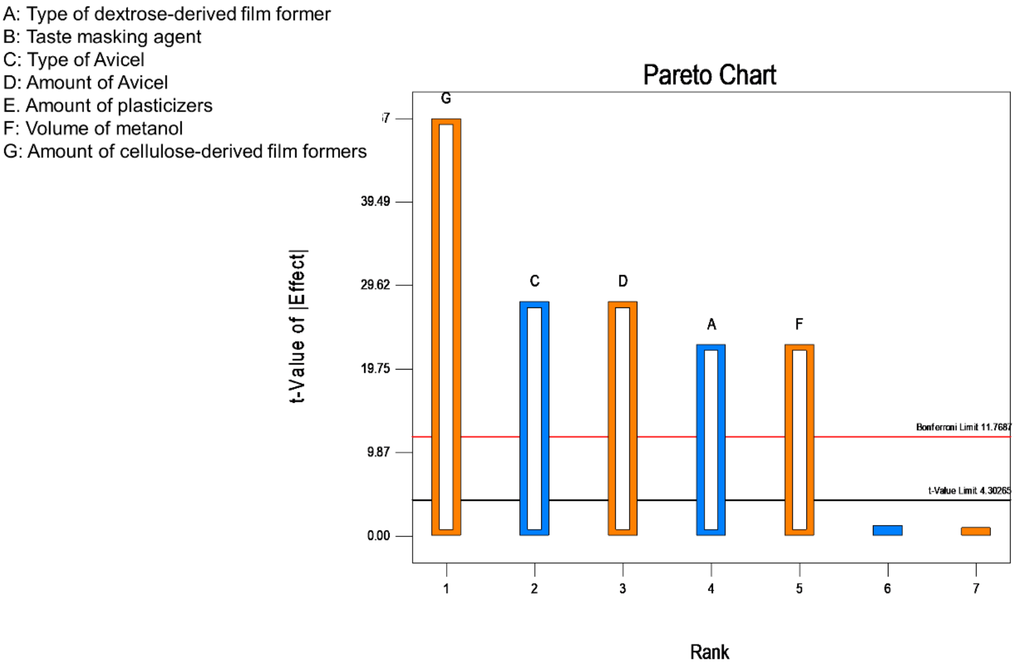

Figure 3. Pareto charts depicting the effect of (A) Type of dextrose-derived film former, (C) Type of Avicel, (D) Amount of Avicel, (F) Volume of methanol and (G) Amount of cellulose-derived film formers on the burst strength of the film (expressed as AUC). Orange colour indicates a positive effect whereas blue colour indicates a negative effect.

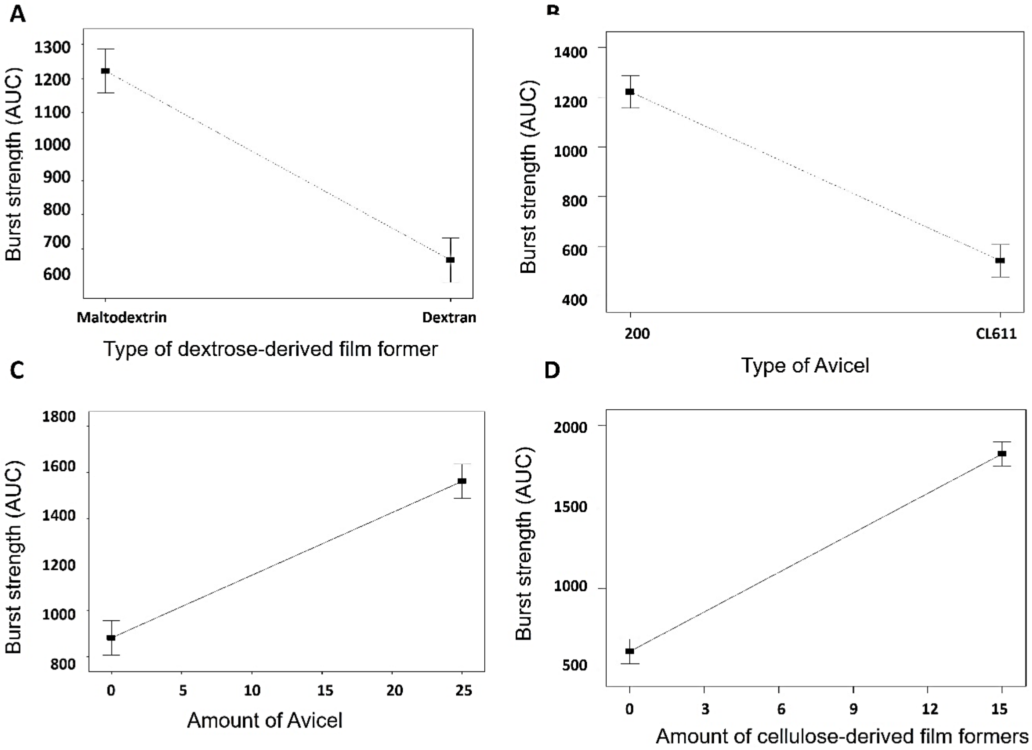

Figure 4. Effect of the significant variables (Film former, Type of Avicel, amount of Avicel and amount of cellulose-derived film formers) on the burst strength expressed as AUC of the film.

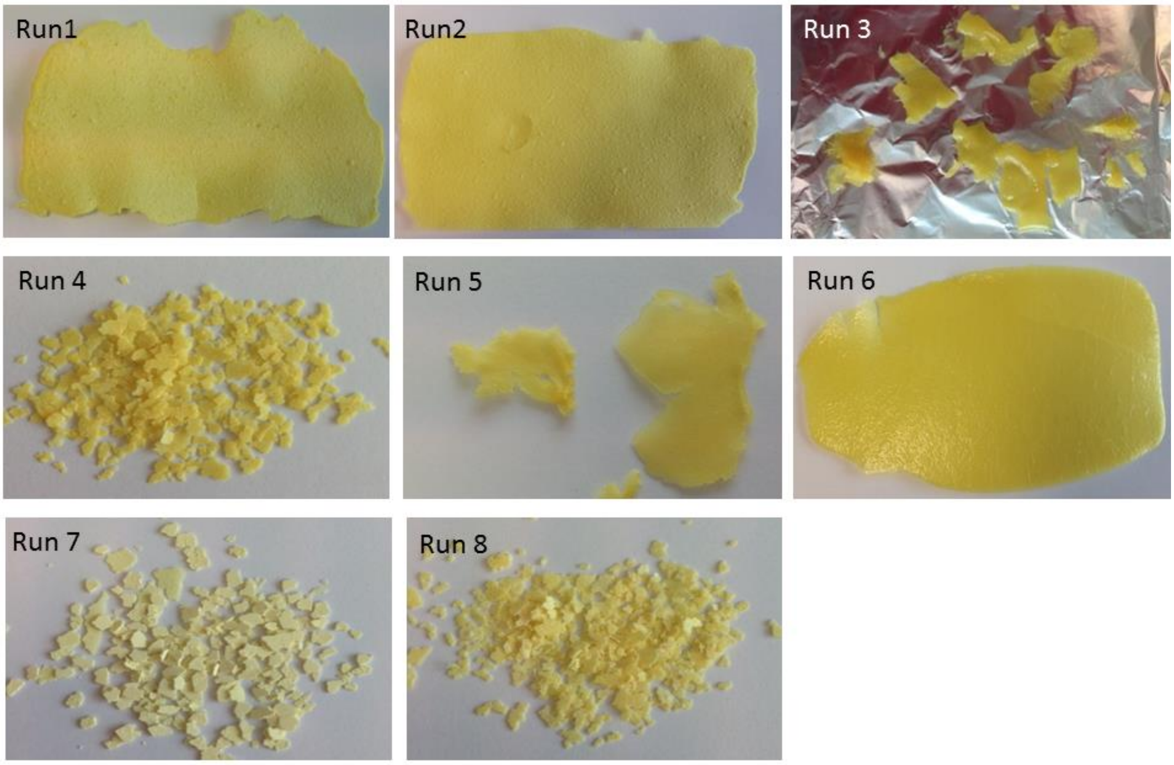

Figure 5. Appearance of the eight AmB-loaded films prepared according to Taguchi matrix design.

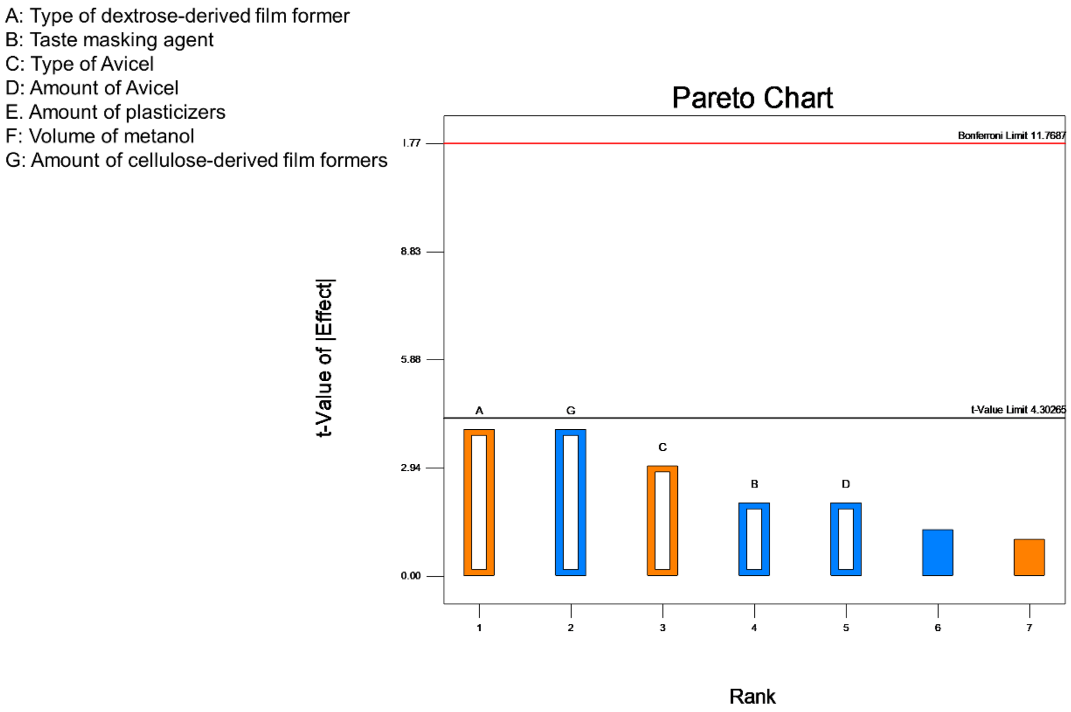

Figure 6. Pareto charts depicting the effect of (A) Type of dextrose-derived film former, (B) Taste masking, (C) Type of Avicel, (D) Amount of Avicel and (G) Amount of cellulose-derived film formers on the appearance of the film. Orange colour indicates a positive effect whereas blue colour indicates a negative effect.

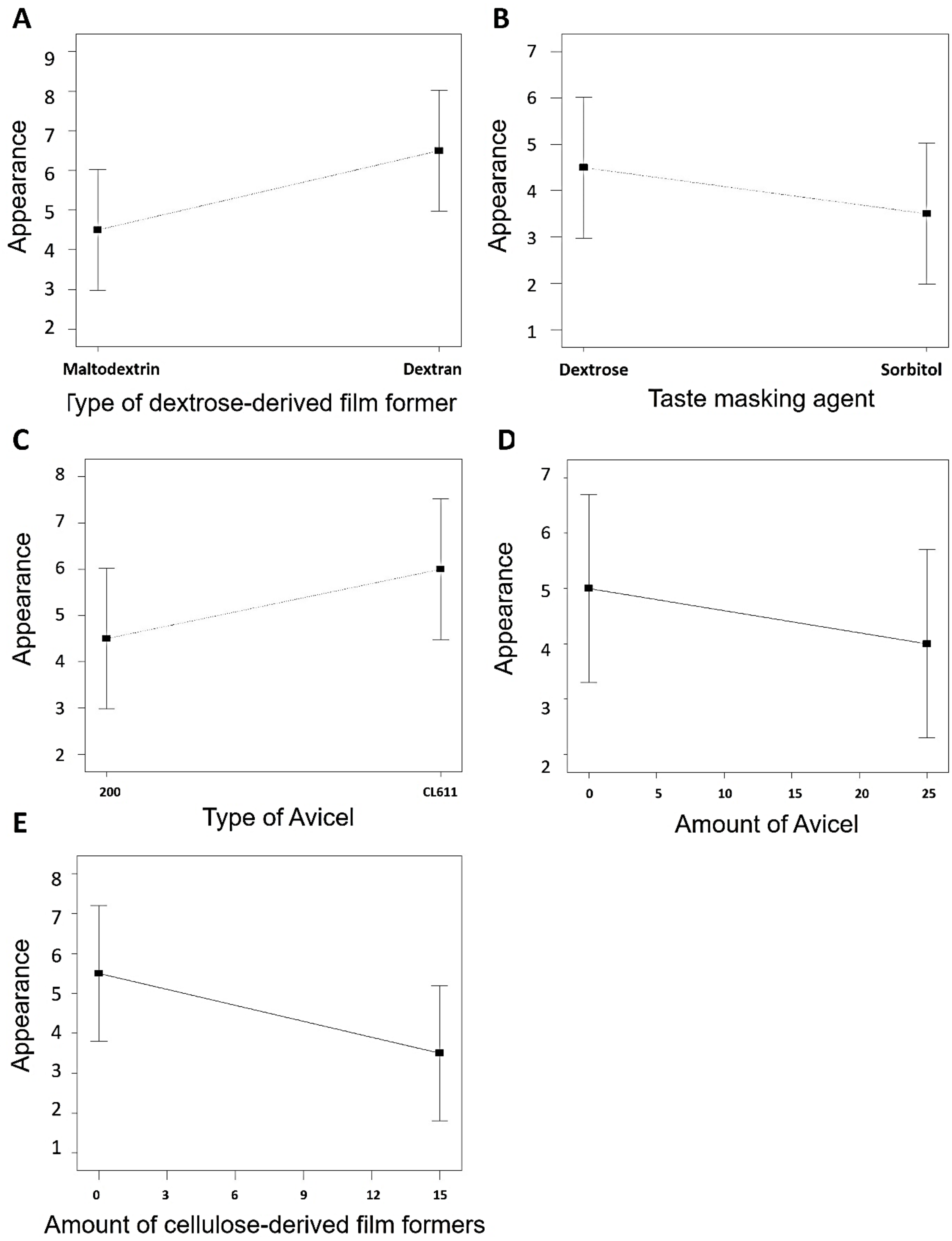

Figure 7. Effect of the five variables with higher impact on the final appearance of the film (Type of Avicel, amount of Avicel, taste masking agent, type of dextrose-derived film former and amount of cellulose derived-film formers).

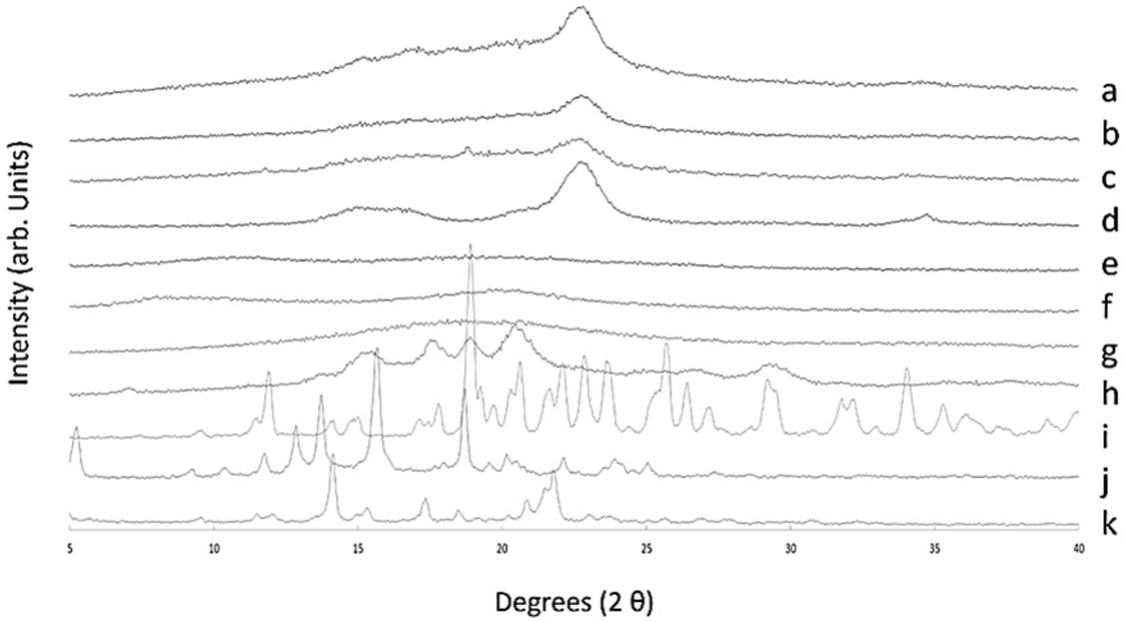

Figure 8. PXRD patterns of raw materials and AmB-loaded ODF before and after DVS analyses. Key: AmB ODF post DVS, b) AmB ODF, c) physical mixture, d) Avicel 200, e) HPMC AS; f) HPC, g) maltodextrin, h) dextran, i) dorbitol, j) dodium deoxycholate, k) AmB.

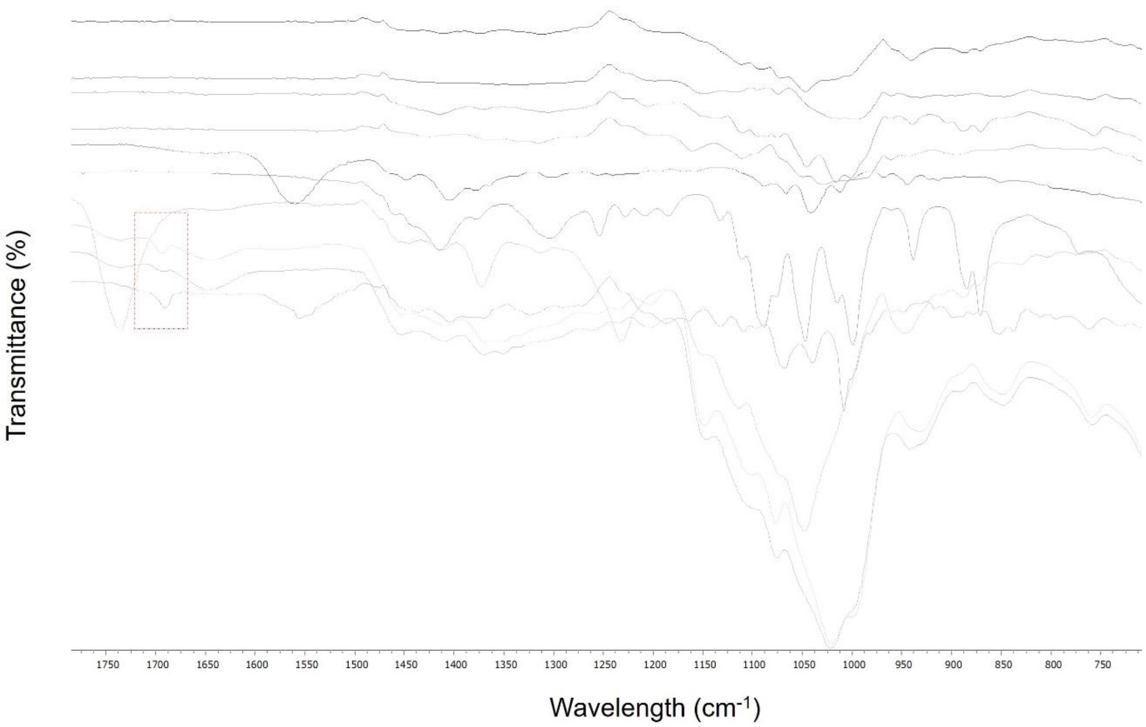

Figure 9. FT-IR spectra. a) HPC, b) maltodextrin, c) dextran, d) Avicel 200, e) sodium deoxycholate, f) sorbitol, g) HPMC 912 AS, h) AmB, i) physical mixture, j) AmB-loaded .
